# QTc prolongation in patients hospitalized in enclosed psychiatric facilities in Corfu

**DOI:** 10.1192/j.eurpsy.2024.997

**Published:** 2024-08-27

**Authors:** P. Argitis, A. Karampas, M. Peyioti, S. Karavia, Z. Chaviaras

**Affiliations:** ^1^Psychiatric, General Hospital of Corfu, Corfu; ^2^Psychiatric, General Hospital of Ioannina, Ioannina, Greece

## Abstract

**Introduction:**

An undeniably significant amount of psychotropic medication can evidently affect the corrected QT (QTc) interval, which puts patients’ lives at risk. More specifically, certain anti-psychotic medication can increase the risk of QTc prolongation and by extension the risk of a potentially fatal arrhythmia or sudden cardiac death.

**Objectives:**

Electrocardiograms (ECG) were contacted in one hundred and four (104) chronic patients, with psychosis, through out their hospitalization in several enclosed psychiatric facilities in Corfu. Almost the entirety of the patients along side their anti-psychotic medication were also taking various other medication for their individual pathological issues. We observed any changes that might have occurred on the ECG in comparison with each patient’s medication and it’s potential effect on the QTc.

**Methods:**

The measurements of the QT interval were made manually in lead V5 and the mathematical conversion was contacted using the Hodges correction formula.

**Results:**

At least one ECG (n = 104) was performed. Among them 29,8% (n=31) had ECG abnormalities, including 13,5% (n=13) with a prolonged Qtc (481.2 ± 26,8 ms). Covariates significantly associated with the QTc were gender (+17.2 ms if female, p < 0.0001) and age (+0.4 ms/year, p = 0.0001).

**Conclusions:**

The QTc prolongation that was evident in a notable number of patients, emphasizes the importance of QTc monitoring in patients who are taking anti-psychotic medication. QTc prolongation risk factors should be assessed before the administration or prescription of any anti-psychotic medication.

**Disclosure of Interest:**

None Declared

